# Effects of high-intensity interval training combined with dietary intervention on body composition, cardiovascular function, endothelial cell function and blood lipid indexes in children with obesity: a randomized controlled trial

**DOI:** 10.3389/fpubh.2025.1698573

**Published:** 2025-11-14

**Authors:** Xinghao Wang, Qiao Meng, Taofeng Liu, Mariusz Lipowski

**Affiliations:** 1 Department of Sport, Gdansk University of Physical Education and Sport, Gdansk, Poland; 2 School of Kinesiology and Physical Education, Zhengzhou University, Zhengzhou, China; 3 Faculty of Social and Humanities, WSB Merito University Gdansk, Gdansk, Poland

**Keywords:** moderate-intensity continuous training, high-intensity interval training, dietary intervention, HIIT combined with dietary intervention on body composition, overweight children

## Abstract

**Objective:**

To investigate the combined effects of moderate-intensity continuous training(MICT), high-intensity interval training (HIIT) and HIIT combined with dietary intervention on body composition, cardiovascular function, and endothelial cell function (as assessed by biomarkers including endothelin-1 and nitric oxide) in overweight children aged 9–12 years with a BMI ≥ 23 kg/m^2^.

**Methods:**

A total of 90 overweight children were randomly assigned into three groups with a 1:1 gender ratio: moderate-intensity continuous training group (MICT, *n* = 30), high-intensity interval training-only group (HIIT-only, *n* = 30), and HIIT combined with dietary intervention group (Joint intervention, *n* = 30). The MICT group underwent a 9-week training program at an intensity of 60–80% of maximal aerobic speed (MAS). The HIIT-only group performed high-intensity interval training at 100–120% of MAS for 9 weeks. The combined intervention group received both HIIT and a diet plan designed by a registered dietitian. Pairwise comparisons were analyzed using the Bonferroni *post hoc* test. Body composition, cardiovascular function, endothelial function, and blood lipid profiles were measured before and after the intervention. Bonferroni post-hoc tests were used for pairwise comparisons to examine the effects of intervention type (MICT, HIIT-only, Joint Intervention) and time (pre- and post-intervention) on each outcome.

**Results:**

After the intervention, all three groups showed significant reductions in body mass index and fat mass. Intergroup comparisons revealed that the Joint Intervention group demonstrated superior improvements in body composition indicators. Both HIIT groups showed greater reductions in body fat percentage compared to the MICT group (*p* < 0.05). The Joint Intervention group exhibited better outcomes in cardiac output (CO) and vasodilatory capacity index (VDC), with values significantly higher than those in the HIIT-only and MICT groups. In contrast, heart rate (HR) and sympathetic nervous response (TCR) were lower in the Joint Intervention group compared to the HIIT-only group, with statistically significant differences (*p* < 0.05). Post-intervention, endothelin-1 (ET-1) and von Willebrand factor (vWF) levels were lower in the Joint Intervention group than in the HIIT-only group. However, flow-mediated dilation (FMD) and nitric oxide (NO) levels were higher in the Joint Intervention group compared to the HIIT-only group, with significant differences (*p* < 0.05). The Joint Intervention group also showed greater improvements in waist circumference, body mass index (BMI), and blood lipid profiles compared to the HIIT-only group, with statistically significant differences (*p* < 0.05).

**Conclusion:**

The combination of high-intensity interval training and dietary intervention promotes fat reduction, enhances antioxidant capacity, and improves cardiorespiratory function in overweight children. This integrated approach effectively improves body mass index, cardiovascular function, and endothelial cell function. The remarkable efficacy of this combined intervention suggests its potential value for clinical application and integration into school-based programs aimed at addressing childhood obesity.

## 1 Introduction

Childhood obesity has emerged as a critical global health crisis, characterized by excess body fat accumulation and adverse cardio-metabolic consequences (1). The prevalence of overweight and obesity among children has increased dramatically over the past three decades, contributing to early-onset insulin resistance, dyslipidemia, endothelial dysfunction, and even subclinical atherosclerosis (2, 3). Epidemiological studies highlight that pediatric obesity not only compromises physical health but also predicts higher all-cause morbidity and mortality in adulthood (4, 5). Therefore, early intervention strategies targeting energy balance, cardiovascular fitness, and endothelial health are essential for mitigating long-term disease risk (6, 7).

Physical exercise is recognized as one of the most effective non-pharmacological interventions for managing obesity and its comorbidities (8). Exercise-induced metabolic adaptations include improved glucose transport, enhanced mitochondrial biogenesis, and optimized lipid oxidation (9, 10). Among exercise modalities, moderate-intensity continuous training (MICT) and high-intensity interval training (HIIT) have attracted extensive research attention.

MICT improves aerobic endurance and cardiorespiratory capacity through sustained submaximal workloads. At the same time, HIIT alternates brief periods of near-maximal exertion with active recovery, inducing greater metabolic and cardiovascular stress within a shorter duration (11). These physiological stimuli enhance oxygen uptake kinetics, cardiac output, and endothelial shear stress, collectively promoting vascular remodeling and increased nitric oxide (NO) bioavailability (12).

The adaptive benefits of exercise are underpinned by several physiological theories. The overload principle posits that biological systems adapt in response to stress exceeding habitual levels, while the specific adaptation to imposed demands (SAID) principle explains the tissue-specific remodeling of cardiac, muscular, and endothelial structures (13).

From a systemic perspective, repeated mechanical and metabolic stress during HIIT triggers endothelial nitric oxide synthase (eNOS) activation, improving vasodilation and reducing total peripheral resistance. This mechanism parallels the adaptive tissue remodeling observed in musculoskeletal rehabilitation models (14). Moreover, the angiogenesis theory supports the notion that exercise stimulates vascular endothelial growth factor (VEGF) pathways, enhancing microvascular density and oxygen delivery to active tissues—critical processes for cardio metabolic improvement in obese youth (15).

Beyond classical cardiovascular mechanisms, recent evidence reveals that exercise also interacts with the gut microbiota metabolism axis (16, 17). Exercise can modulate microbial diversity and short-chain fatty acid production, thereby influencing systemic inflammation and energy utilization (18). This bidirectional regulation between muscular activity and intestinal homeostasis represents a novel theoretical pathway linking physical activity to improved metabolic flexibility and body composition regulation in children with obesity.

Energy intake remains the other crucial determinant of metabolic balance. Nutritional strategies emphasizing macronutrient redistribution, moderate carbohydrates, balanced proteins, and reduced fat intake can optimize substrate utilization during exercise and recovery (19). Combining dietary control with HIIT provides a dual regulatory mechanism, wherein exercise enhances lipid mobilization and mitochondrial function, while diet constrains caloric excess and supports improved insulin sensitivity (20). The energy compensation theory suggests that concurrent dietary control prevents post-exercise hyperphagia and optimizes net energy expenditure (21). Hence, a combined exercise–dietary model may achieve superior outcomes in both weight regulation and vascular health.

Pediatric exercise prescription must adhere to developmental and safety frameworks. The Youth Physical Development Model (YPD) emphasizes that training programs for children should prioritize movement competence, motor control, and progressive overload appropriate to their growth stage (22). HIIT protocols, when properly supervised, can safely improve cardiovascular and muscular performance in school-aged children by stimulating central and peripheral adaptations without excessive strain (23). Such structured interventions have shown high adherence and feasibility in youth populations, making them suitable for school- or community-based applications.

Although numerous studies have examined exercise interventions for pediatric obesity, direct comparisons among MICT, HIIT, and HIIT combined with dietary intervention remain scarce. Moreover, limited research has concurrently assessed body composition, cardiovascular function, endothelial biomarkers, and lipid metabolism within a single, controlled framework. Addressing these gaps, the present study examines the comparative effects of three intervention modalities, MICT, HIIT, and combined HIIT plus dietary regulation, on multidimensional health outcomes in overweight children.

We hypothesize that the combined intervention would elicit the most pronounced improvements in body composition, cardiovascular performance, endothelial function, and blood lipid regulation, reflecting a synergistic interaction between exercise-induced physiological adaptation and dietary modulation.

## 2 Methods

### 2.1 Procedure

A total of 90 overweight children (45 boys and 45 girls, aged 9–12 years) hospitalized at our institution between January 2024 and December 2024 were included in this study. Participants were randomly assigned using random numbers generated by SPSS 22.0 software. Based on the “sealed envelope method,” they were divided in a 1:1 gender ratio into three groups: moderate-intensity continuous training (MICT), high-intensity interval training (HIIT), and a combined intervention group (HIIT + dietary intervention). Each group contained 30 children (15 boys and 15 girls). To minimize measurement bias, assessors were blinded to group allocation. The mean age was 10.52 ± 0.75 years in the MICT group, 10.71 ± 0.92 years in the HIIT group, and 10.69 ± 0.95 years in the combined intervention group. No statistically significant differences were observed among the three groups in terms of gender distribution or age (*p* > 0.05).

Inclusion Criteria: Met the diagnostic criteria for childhood obesity as defined in the Survey of the Prevalence of Overweight and Obesity Among Children and Adolescents in China (22). Had not taken any weight-loss medication in the past 3 months. Had no congenital metabolic abnormalities. Had a body mass index (BMI) ≥ 23 kg/m^2^. The children and their families were required to provide signed informed consent. The study protocol was approved by the hospital ethics committee.

Exclusion Criteria: Children with severe cardiovascular or endocrine diseases. Children who engaged in regular exercise or had taken weight-loss medication within the last 3 months. Children with psychiatric disorders. Children with severe cardiac, pulmonary, hepatic, or renal dysfunction. These criteria were applied to screen eligible children to ensure the feasibility and accuracy of the study.

*A priori* power analysis (G*Power 3.1) indicated that 78 participants were required to detect a medium effect (*f* = 0.25, *α* = 0.05, 1 − *β* = 0.80). The current sample (*n* = 90) yields *post hoc* power >0.75, ensuring adequate detection of moderate-to-large effects.

### 2.2 Treatment method

All participants underwent running-based exercise interventions on an outdoor athletic track. Before the formal training period, a two-week adaptive training regimen consisting of four sessions per week was implemented (24).

The moderate-intensity continuous training (MICT) group engaged in 30-min sessions per exercise bout, with intensity maintained at 60–80% of maximal aerobic speed (MAS).

The high-intensity interval training (HIIT) group followed a structured program comprising four sessions per week (Monday, Wednesday, Friday, and Saturday) over 9 weeks. Each session began with a 15-min jogging warm-up to reduce injury risk (24) followed by HIIT exercises aimed at enhancing cardiopulmonary function. The HIIT protocol included a 6-min sprint interval (20 m at maximal effort) and a 30-s all-out sprint performed at 100–120% MAS, interspersed with 30-s active recovery jogs at 50% MAS. This high-low intensity cycle was repeated 10 times, with 5-min rest intervals between sets. Each child’s MAS was individually determined before the intervention using the standardized 20-m shuttle run test (Léger test). Sessions concluded with a 10-min cool-down period involving light jogging and stretching to promote recovery.

The combined intervention group followed the same HIIT protocol while also adhering to a personalized dietary plan. Daily nutritional intake was calculated using the Schofield equation, based on each child’s age, body weight, and height. The macronutrient distribution was set at 55% carbohydrates, 25% fat, and 20% protein. A registered dietitian developed standardized meal plans for each participant, provided ongoing supervision of the dietary regimen, and delivered weekly consultations, standardized meal plans, and family nutrition education to ensure compliance. Parents were instructed to strictly monitor their children’s adherence to the meal plan. Implementation was monitored using food diaries and 24-h dietary recalls. All three exercise groups were supervised by certified instructors during training sessions, which were conducted on the school athletic track. The combined group additionally received oversight from a dietitian to ensure effective integration of exercise and dietary modifications.

### 2.3 Observation indexes

Body composition indicators, in addition to height, weight, and body mass index (BMI), included body fat, body fat percentage, lean body mass, and visceral adipose tissue, measured via dual-energy X-ray absorptiometry (DXA; Lunar Prodigy, GE Healthcare, United States). The device was operated by hospital physicians. Prior to testing, participants were required to fast and wear light close-fitting clothing without metal accessories such as rings or necklaces. Measurements were taken in a supine position using the standard DXA mode.

Cardiovascular function was assessed using the ZXG-G Automatic Cardiovascular Diagnostic System (Manufacturer: Xinda Medical; Model: ZXG-G-2020), a device validated for pediatric populations with 98% sensitivity and 95% specificity. After inputting each child’s height, waist circumference, and blood pressure into the system, a sensor was securely attached to the radial artery to capture stabilized pulse waveforms. This allowed for the calculation of heart rate (HR), cardiac output (CO), total circumferential resistance (TCR), and vasodilation coefficient (VDC).

For biochemical analysis, fasting venous blood samples (3 mL) were collected from all participants before and after the 9-week intervention. Blood was centrifuged at 3,000 rpm for 10 min using a centrifuge with a 5-cm radius, and plasma aliquots were stored at −80 °C. Endothelin-1 (ET-1), von Willebrand factor (vWF), flow-mediated dilation (FMD), nitric oxide (NO), and lipid profiles (HDL-C, LDL-C, TC, TG) were quantified using commercially available ELISA kits (R&D Systems, Cat. No. DET100/DWF200). The assay protocol involved sample dilution with inclusion buffer, incubation at 37 °C for 4 h, and three washes to remove unbound substances. Antigen–antibody complexes were formed, followed by the addition of enzyme-labeled antibodies and colorimetric substrate development. Concentrations were determined based on optical density readings.

Anthropometric measurements included waist circumference, hip circumference, and BMI. Waist and hip circumferences were measured using a flexible tape, while height and weight were recorded using a stadiometer and calibrated scale. BMI was calculated as weight (kg) divided by height squared (m^2^). Age- and sex-adjusted BMI z-scores were derived using the World Health Organization (WHO) Growth Reference Standards to account for pediatric growth patterns. All measurements were performed by trained staff to ensure consistency and accuracy. All assessments were conducted twice by the same operator. Intra-class correlation coefficients (ICCs) exceeded 0.90 for DXA and 0.88 for cardiovascular measures, indicating excellent reliability (Table 1).

**Table 1:** Demographics (*n* = 90).

Variable	Total (*n* = 90)	MICT (*n* = 30)	HIIT-only (*n* = 30)	Joint intervention (*n* = 30)
Boys/girls (n)	45/45	15/15	15/15	15/15
Age (years)	10.64 ± 0.86	10.52 ± 0.75	10.71 ± 0.92	10.69 ± 0.95
Height (m)	1.48 ± 0.06	1.48 ± 0.08	1.47 ± 0.09	1.49 ± 0.07
Body mass index (kg/m^2^)	24.36 ± 1.53	24.31 ± 0.95	24.25 ± 1.05	24.45 ± 1.99

### 2.4 Statistical methods

Data analysis was performed using SPSS 22.0 software. An independent samples t-test was used to compare measures between groups, while changes in data before and after the intervention were analyzed using repeated measures analysis of variance (ANOVA). Results with *p*-values less than 0.05 were considered statistically significant.

## 3 Results

### 3.1 Comparison of body composition indicators

**Figure 1:**
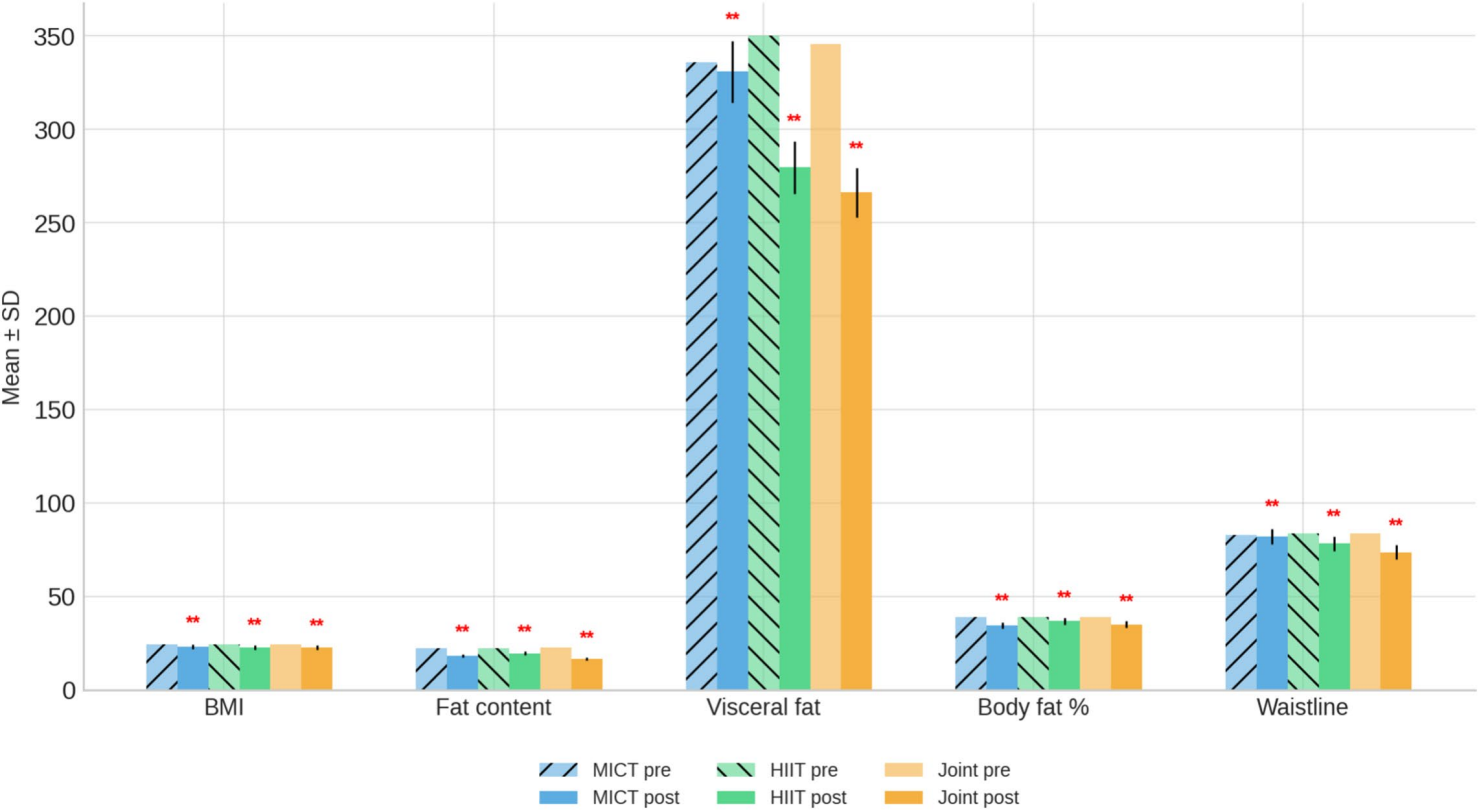
Body composition bar chart before and after intervention. **p < 0.01, compared with the same group before intervention.

After the 9-week intervention, all three groups showed improvements in body-composition indicators compared with their baseline values (Table 2 and Figure 1).

**Table 2:** Changes in body composition before and after subject intervention (*n* = 90).

Variable	MICT (*n* = 30)		HIIT (*n* = 30)		Joint intervention group (*n* = 30)	
	Pre-intervention	Post-intervention	pre-intervention	Post-intervention	Pre-intervention	Post-intervention
BMI (kg/m^2^)	24.41 ± 0.24	23.22 ± 0.44**	24.38 ± 0.41	22.68 ± 0.42**	24.41 ± 0.39	22.64 ± 0.78**
fat content (kg)	22.34 ± 1.13	18.28 ± 0.97**	22.18 ± 1.18	19.46 ± 0.93**	22.62 ± 1.10	16.39 ± 1.0.4** ^£^
visceral fat (g)	336.10 ± 39.93	330.83 ± 31.87	350.00 ± 36.56	279.70 ± 14.17**	345.77 ± 40.46	266.20 ± 12.77**
Body fat percentage (%)	38.97 ± 2.20	34.43 ± 3.10**	38.91 ± 2.35	36.82 ± 2.43**	39.06 ± 2.02	34.96 ± 2.17** ^£^
waistline (cm)	82.97 ± 2.18	82.15 ± 3.39**	83.54 ± 3.16	78.20 ± 2.61**	83.50 ± 3.30	73.70 ± 3.09** ^£^

^*^P < 0.05, ^**^P < 0.01, compared with the same group before intervention; ^#^*p* < 0.05, compared with the MICT group; ^£^*p* < 0.05, compared with the HIIT group.

Body mass index (BMI): Significant reductions were observed in all groups (*p* < 0.01). The MICT, HIIT, and Joint Intervention groups decreased from 24.41 ± 0.24 to 23.22 ± 0.44, 24.38 ± 0.41 to 22.68 ± 0.42, and 24.41 ± 0.39 to 22.64 ± 0.78 kg/m^2^, respectively. The magnitude of decrease was greatest in the Joint Intervention group; Total fat mass: All groups showed significant decreases in fat mass post-intervention (*p* < 0.01). The mean reduction was most pronounced in the Joint Intervention group (−6.23 kg), compared with −4.06 kg in the MICT group and −2.72 kg in the HIIT group; Visceral fat: A significant decline occurred only in the HIIT-based groups (*p* < 0.01), decreasing from 350.00 ± 36.56 to 279.70 ± 14.17 g in the HIIT group and from 345.77 ± 40.46 to 266.20 ± 12.77 g in the Joint Intervention group, whereas the MICT group showed a minimal change (336.10 ± 39.93–330.83 ± 31.87 g); Body fat percentage: All three interventions significantly lowered body-fat percentage (*p* < 0.01). The Joint Intervention group had a reduction of approximately 4 percentage points (39.06 ± 2.02–34.96 ± 2.17%), surpassing the MICT and HIIT groups; Waist circumference: Waistline measurements declined in each group (*p* < 0.01). The greatest improvement was recorded in the Joint Intervention group (83.50 ± 3.30–73.70 ± 3.09 cm), followed by HIIT (83.54 ± 3.16–78.20 ± 2.61 cm) and MICT (82.97 ± 2.18–82.15 ± 3.39 cm).

Overall, the combination of HIIT plus dietary intervention produced the most substantial improvements across all adiposity-related variables, indicating a synergistic effect of exercise and dietary regulation on fat reduction and central adiposity control in overweight children.

### 3.2 Comparison of cardiovascular function indexes

**Figure 2:**
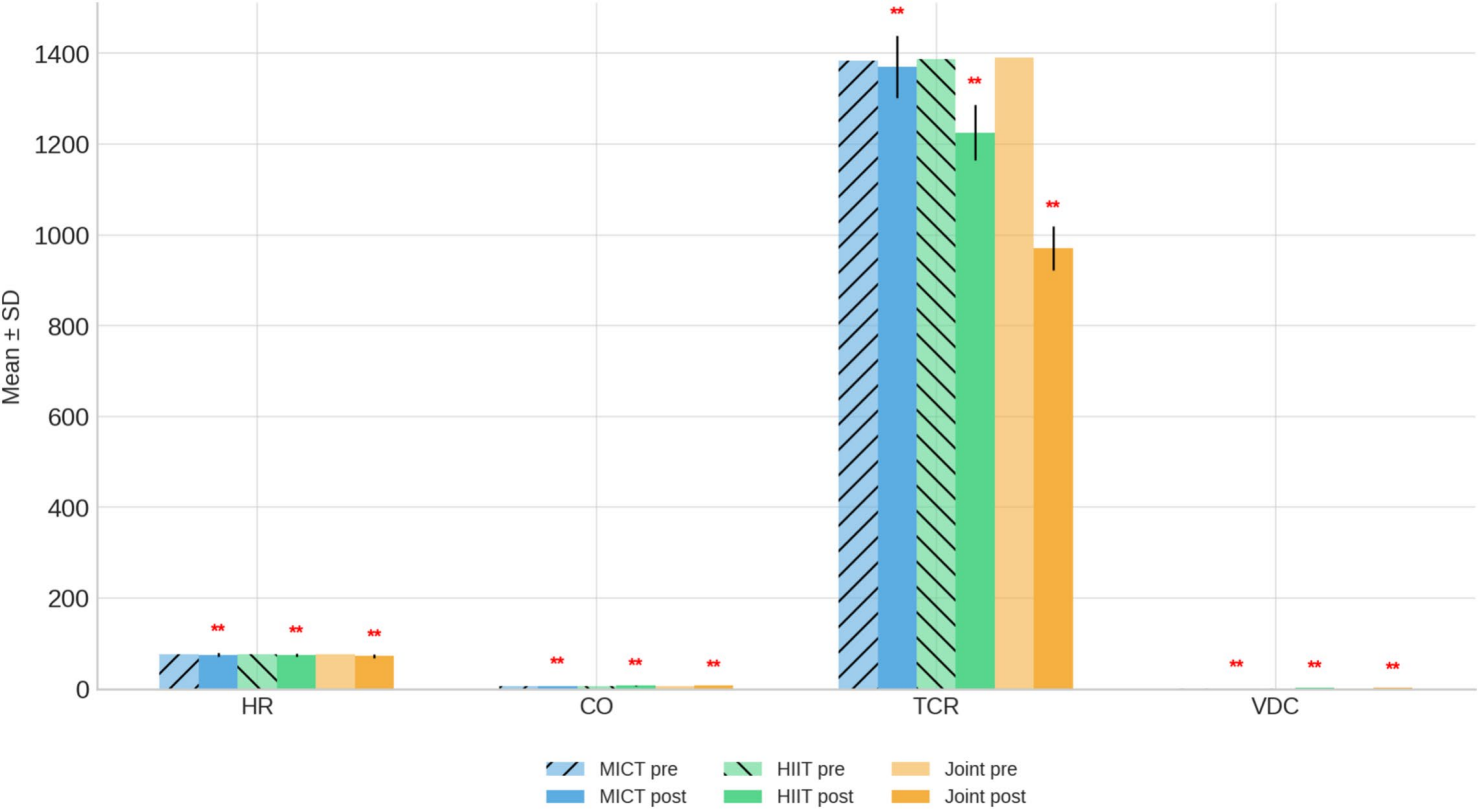
Cardiovascular function bar chart before and after intervention. **p < 0.01, compared with the same group before intervention.

After the 9-week intervention, all three groups exhibited varying degrees of cardiovascular improvement (Table 3 and Figure 2).

**Table 3:** Changes in cardiovascular function before and after subject intervention (*n* = 90).

Variable	MICT (*n* = 30)		HIIT (*n* = 30)		Joint intervention group (*n* = 30)	
	Pre-intervention	Post-intervention	Pre-intervention	Post-intervention	Pre-intervention	Post-intervention
HR (number /min)	76.18 ± 1.86	75.49 ± 1.46**	76.68 ± 1.87	74.38 ± 1.36**	75.80 ± 1.80	72.40 ± 1.99** ^£^
CO [V/(L•min)]	5.50 ± 1.15	6.41 ± 1.26**	5.82 ± 1.12	7.12 ± 1.01**	5.79 ± 1.10	8.23 ± 1.11** ^£^
TCR (dyn•s•cm^5^)	1384.07 ± 19.46	1369.99 ± 14.98**	1387.76 ± 19.73	1225.70 ± 60.57**	1390.67 ± 20.77	971.39 ± 8.99** ^£^
VDC index	1.25 ± 0.17	1.34 ± 0.24	1.27 ± 0.19	2.44 ± 0.38** ^#^	1.27 ± 0.19	3.44 ± 0.41** ^£^

^*^P < 0.05, ^**^P < 0.01, compared with the same group before intervention; ^#^*p* < 0.05, compared with the MICT group; ^£^*p* < 0.05, compared with the HIIT group.

Heart Rate (HR): All groups showed a significant decrease in resting HR following the intervention (*p* < 0.01). The MICT group declined slightly from 76.18 ± 1.8 to 75.49 ± 1.46 beats/min, while greater reductions were observed in the HIIT (76.68 ± 1.87–74.38 ± 1.36 beats/min) and Joint Intervention groups (75.80 ± 1.80–72.40 ± 1.99 beats/min). The reduction magnitude was most pronounced in the Joint Intervention group, indicating enhanced cardiac efficiency; Cardiac Output (CO): All interventions significantly improved CO (*p* < 0.01). The MICT group increased from 5.50 ± 1.15 to 6.41 ± 1.26 L/min, the HIIT group from 5.82 ± 1.12 to 7.12 ± 1.01 L/min, and the Joint Intervention group from 5.79 ± 1.10 to 8.23 ± 1.11 L/min. The combined program yielded the greatest enhancement, suggesting synergistic effects of high-intensity exercise and dietary regulation on cardiac performance; Total Circumferential Resistance (TCR): Systemic vascular resistance decreased significantly in all groups (*p* < 0.01). TCR declined from 1384.07 ± 19.46 to 1369.99 ± 14.98 dyn·s·cm^−5^ in the MICT group, 1387.76 ± 19.73–1225.70 ± 60.57 in the HIIT group, and 1390.67 ± 20.77–971.39 ± 8.99 in the Joint Intervention group. The marked reduction in the combined group suggests improved peripheral vascular compliance and decreased afterload; Vasodilation Capacity (VDC): The VDC index improved substantially in the HIIT and Joint Intervention groups (*p* < 0.01), rising from 1.27 ± 0.19 to 2.44 ± 0.38 and 3.44 ± 0.41, respectively, while no significant change occurred in the MICT group.

Overall, both HIIT and the combined HIIT + dietary intervention produced pronounced enhancements in cardiac output and vascular function compared with MICT alone. The largest gains in CO and VDC, alongside the greatest reduction in TCR, were observed in the Joint Intervention group, indicating superior cardiovascular adaptation and endothelial regulation when exercise intensity and dietary control are integrated.

### 3.3 Comparison of endothelial cell function indexes

**Figure 3:**
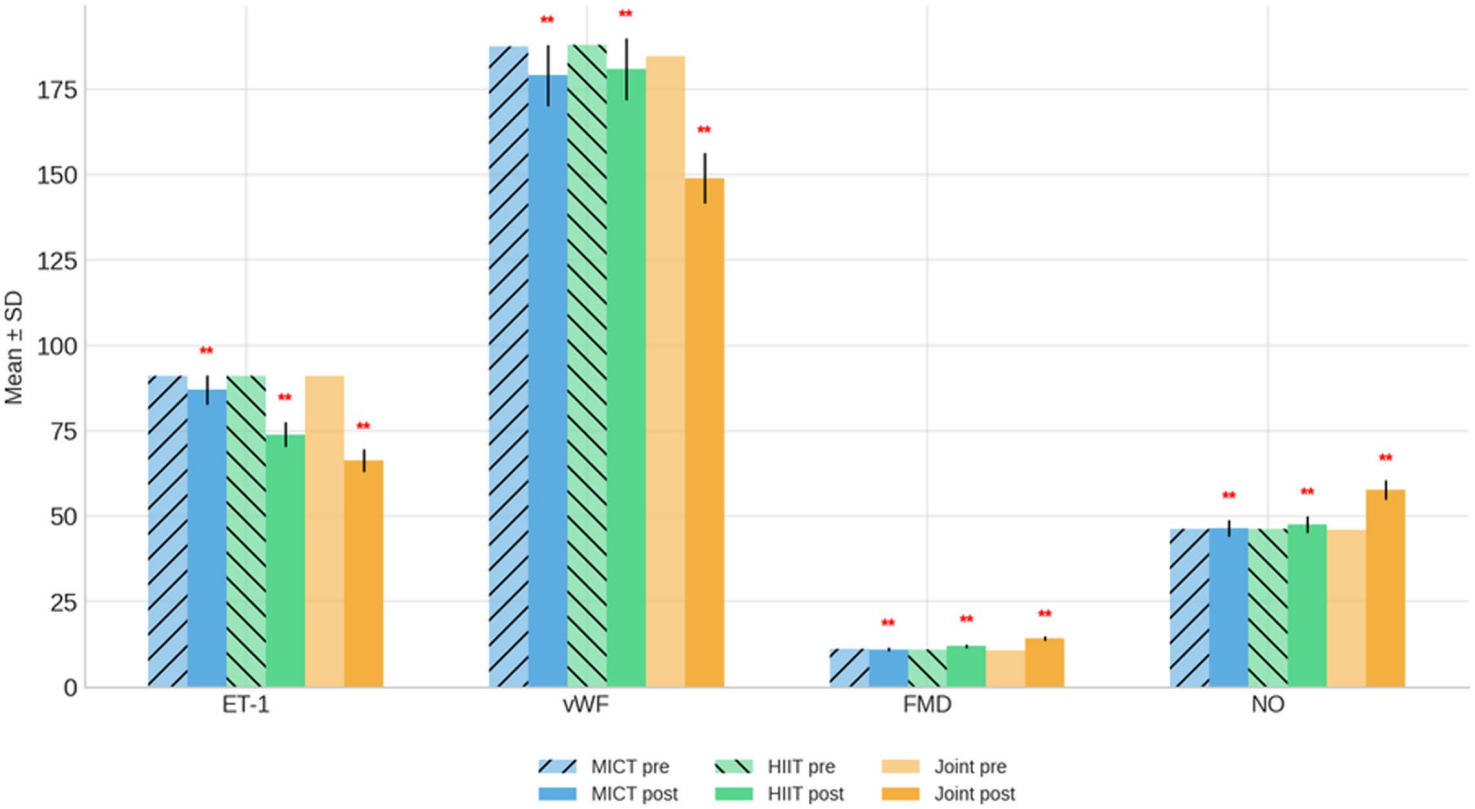
Endothelial cell function bar chart before and after intervention. **p < 0.01, compared with the same group before intervention.

Following the 9-week intervention, indices of endothelial function demonstrated significant improvements, particularly in the HIIT and Joint Intervention groups (Table 4 and Figure 3).

**Table 4:** Changes in endothelial cell function before and after subject intervention (*n* = 90).

Variable	MICT (*n* = 30)		HIIT (*n* = 30)		Joint intervention group (*n* = 30)	
	Pre-intervention	Post-intervention	Pre-intervention	Post-intervention	Pre-intervention	Post-intervention
ET-1 (ng/L)	91.02 ± 1.37	86.99 ± 2.00**	91.12 ± 1.56	73.93 ± 1.83** ^#^	91.14 ± 1.24	66.43 ± 1.99** ^£^
vWF (%)	187.48 ± 9.18	179.08 ± 6.12**	187.89 ± 8.75	180.81 ± 6.38**	184.69 ± 7.99	148.95 ± 5.05** ^£^
FMD (%)	11.09 ± 1.53	11.00 ± 1.29	11.05 ± 1.38	11.98 ± 1.20**	10.70 ± 1.54	14.24 ± 1.32** ^£^
NO (^mol/L)	46.34 ± 4.79	46.50 ± 4.46	46.27 ± 3.81	47.63.86 ± 4.21	46.00 ± 4.27	57.74 ± 2.62** ^£^

^*^P < 0.05, ^**^P < 0.01, compared with the same group before intervention; ^#^*p* < 0.05, compared with the MICT group; ^£^*p* < 0.05, compared with the HIIT group.

Endothelin-1 (ET-1): Plasma ET-1 levels decreased significantly in all three groups (*p* < 0.01), indicating reduced vasoconstrictive activity. The reduction was modest in the MICT group (91.02 ± 1.37–86.99 ± 2.00 ng/L) but markedly greater in the HIIT (91.12 ± 1.56–73.93 ± 1.83 ng/L) and Joint Intervention groups (91.14 ± 1.24–66.43 ± 1.99 ng/L). The greatest decline in ET-1 occurred in the combined program, reflecting enhanced vascular homeostasis; Von Willebrand Factor (vWF): Similar trends were observed for vWF, a marker of endothelial activation. Levels decreased significantly in all groups (*p* < 0.01), with the most pronounced improvement seen in the Joint Intervention group (184.69 ± 7.99–148.95 ± 5.05%), followed by MICT and HIIT. These reductions suggest decreased endothelial stress and improved vascular integrity; Flow-Mediated Dilation (FMD): FMD improved significantly in the HIIT and Joint Intervention groups (*p* < 0.01), while remaining stable in the MICT group. Increases were observed from 11.05 ± 1.38 to 11.98 ± 1.20% in HIIT and from 10.70 ± 1.54 to 14.24 ± 1.32% in the Joint Intervention group, indicating superior endothelial responsiveness and vasodilatory capacity after high-intensity or combined interventions; Nitric Oxide (NO): Serum NO concentrations, reflecting endothelial nitric oxide synthase activity, rose markedly in the Joint Intervention group (46.00 ± 4.27–57.74 ± 2.62 μmol/L; *p* < 0.01), with modest increases in the HIIT group and no significant change in MICT. The substantial elevation of NO further corroborates improved endothelial function.

Collectively, these results demonstrate that HIIT and especially HIIT combined with dietary intervention elicited greater endothelial benefits than MICT alone. The combined intervention achieved the largest reductions in ET-1 and vWF, alongside significant gains in FMD and NO levels, suggesting enhanced vasodilatory efficiency and reduced vascular inflammation.

### 3.4 Comparison of blood lipid indexes

**Figure 4:**
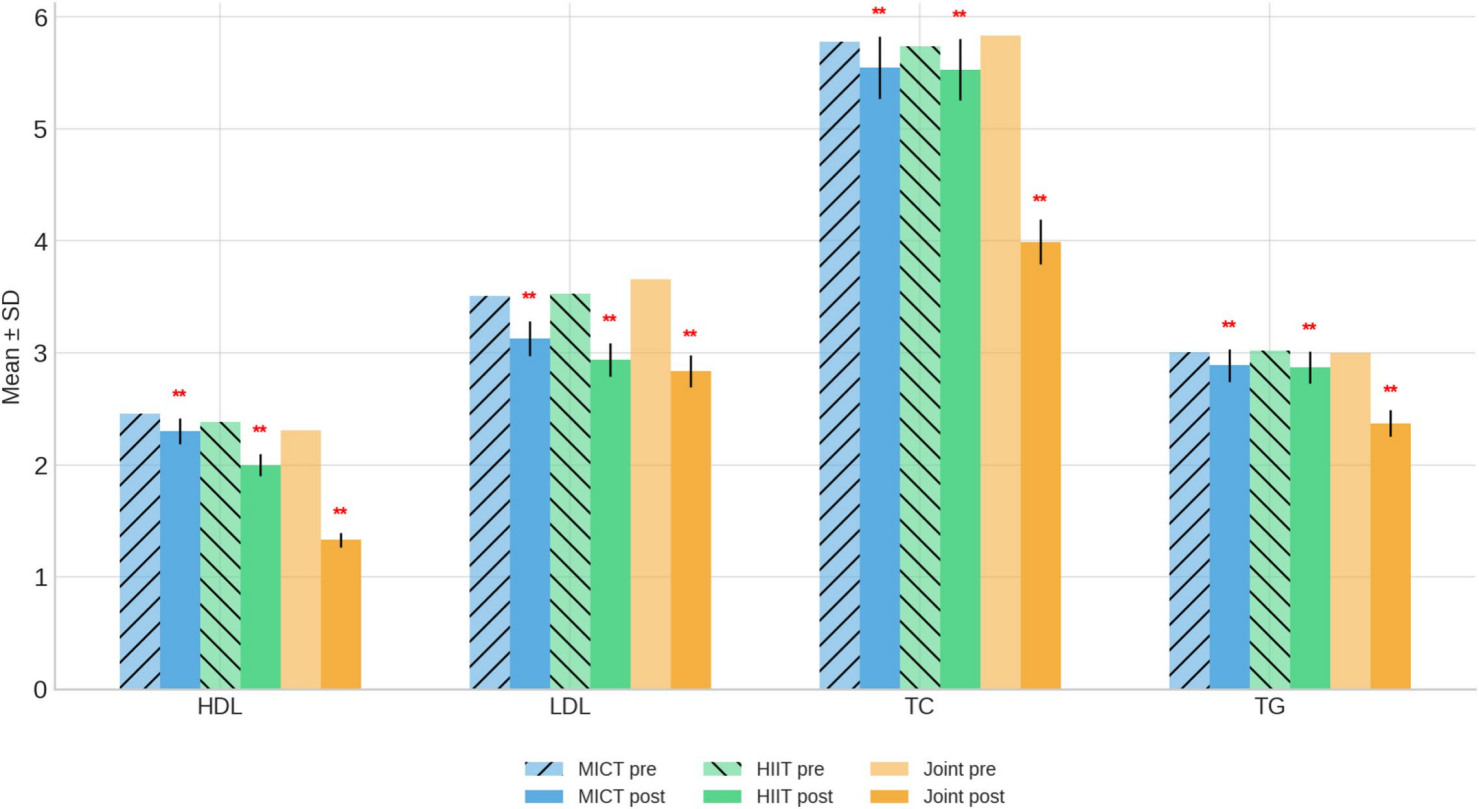
Blood lipid indexes bar chart before and after intervention. **p < 0.01, compared with the same group before intervention.

After 9 weeks of intervention, all three groups showed varying degrees of improvement in blood lipid parameters, with the most pronounced effects observed in the Joint Intervention group (Table 5 and Figure 4).

**Table 5:** Changes in blood lipid indexes before and after subject intervention (*n* = 90).

Variable	MICT (*n* = 30)		HIIT (*n* = 30)		Joint intervention group (*n* = 30)	
	Pre-intervention	Post-intervention	Pre-intervention	Post-intervention	Pre-intervention	Post-intervention
HDL (mmol/L)	2.46 ± 0.38	2.30 ± 0.34**	2.38 ± 0.29	2.00 ± 0.29** ^#^	2.31 ± 0.20	1.33 ± 0.32** ^£^
LDL (mmol/L)	3.51 ± 0.83	3.13 ± 0.53**	3.53 ± 0.68	2.94 ± 0.50** ^#^	3.66 ± 0.52	2.84 ± 0.51** ^£^
TC (mmol/L)	5.78 ± 0.89	5.55 ± 0.80	5.74 ± 0.76	5.53 ± 0.80**	5.83 ± 0.85	3.99 ± 1.08** ^£^
TG (mmol/L)	3.01 ± 0.26	2.89 ± 0.29	3.02 ± 0.26	2.87 ± 0.13*	3.00 ± 0.23	2.37 ± 0.17^** £^

^*^P < 0.05, ^**^P < 0.01, compared with the same group before intervention; ^#^*p* < 0.05, compared with the MICT group; ^£^*p* < 0.05, compared with the HIIT group.

High-Density Lipoprotein Cholesterol (HDL-C): HDL-C levels significantly decreased in all groups (*p* < 0.01). Values declined from 2.46 ± 0.38 to 2.30 ± 0.34 mmol/L in the MICT group, from 2.38 ± 0.29 to 2.00 ± 0.29 mmol/L in the HIIT group, and from 2.31 ± 0.20 to 1.33 ± 0.32 mmol/L in the Joint Intervention group. Although HDL-C typically plays a protective role, the concurrent reduction of total cholesterol and LDL-C suggests an overall favorable lipid redistribution rather than an adverse change; Low-Density Lipoprotein Cholesterol (LDL-C): LDL-C decreased significantly in all groups (*p* < 0.01). The MICT group declined from 3.51 ± 0.83 to 3.13 ± 0.53 mmol/L, the HIIT group from 3.53 ± 0.68 to 2.94 ± 0.50 mmol/L, and the Joint Intervention group from 3.66 ± 0.52 to 2.84 ± 0.51 mmol/L. The combined program produced the largest reduction, reflecting improved lipid metabolism and reduced cardiovascular risk; Total Cholesterol (TC): TC decreased modestly in the MICT and HIIT groups (*p* < 0.05) and markedly in the Joint Intervention group (*p* < 0.01). The mean TC reduction in the combined group (from 5.83 ± 0.85 to 3.99 ± 1.08 mmol/L) indicates strong synergistic effects between HIIT and dietary control on lipid regulation; Triglycerides (TG): TG levels decreased across all groups, with significance reached in the HIIT (*p* < 0.05) and Joint Intervention groups (*p* < 0.01). The Joint Intervention group achieved the greatest decline (from 3.00 ± 0.23 to 2.37 ± 0.17 mmol/L), demonstrating enhanced fat oxidation and improved lipid clearance.

Overall, all interventions improved lipid metabolism, but HIIT combined with dietary modification yielded the most favorable lipid profile, characterized by pronounced reductions in LDL-C, TC, and TG levels. These findings underscore the combined intervention’s superior efficacy in mitigating dyslipidemia associated with pediatric obesity.

## 4 Discussion

It is well-established that prolonged moderate-intensity continuous training (MICT) can reduce body fat and improve body composition (25, 26). This study further demonstrates that short-term high-intensity interval training (HIIT) can produce similar effects. The results confirm that a 9-week combined intervention incorporating HIIT and dietary modifications significantly improves cardiovascular function, endothelial health, body composition, and blood lipid profiles in overweight children, compared to either MICT or HIIT alone. These findings highlight the synergistic effect of structured exercise and nutritional regulation, offering a practical solution to address childhood obesity.

### 4.1 Comparative efficacy and pattern of response

Our results revealed that the combined intervention group exhibited superior improvements in cardiac output (CO), vasodilatation coefficient (VDC), and nitric oxide (NO) levels, alongside greater reductions in waist circumference, BMI, and LDL cholesterol. The enhanced CO and VDC values suggest improved cardiovascular efficiency, likely attributable to HIIT-induced cardiac adaptation and dietary modulation of vascular resistance. Elevated NO levels and reduced endothelin-1 (ET-1) further indicate better endothelial function, potentially driven by exercise-mediated increases in shear stress and diet-induced attenuation of low-grade inflammation and oxidative stress (27, 28). Improved endothelial function likely stems from increased nitric oxide synthase activity and reduced oxidative stress (29). Notably, the significant reduction in waist circumference and BMI aligns with prior studies emphasizing the role of HIIT in visceral fat mobilization and dietary control in energy balance regulation. Taken together, the response profile across central adiposity, hemodynamics, and endothelial biomarkers supports a coherent, systems-level adaptation favoring improved cardio metabolic health.

### 4.2 Consistency with and extension of prior literature

Our findings corroborate recent pediatric-focused research. For instance, Mu et al. reported similar BMI reductions in obese children following aerobic exercise and low-carbohydrate diets, while Liu et al. observed improved lipid profiles with combined interventions (30, 31). However, our study extends these findings by demonstrating specific enhancements in endothelial markers (e.g., FMD, NO) rarely explored in pediatric cohorts. The HIIT protocol’s safety and efficacy in children also align with Eddolls, W. T et al., who documented metabolic benefits without adverse effects in adolescents (25, 32). By integrating vascular endpoints with body composition and lipids, our trial adds mechanistic plausibility to the clinical benefits of combined lifestyle therapy during late childhood.

### 4.3 Mechanistic interpretation: an integrated model

The superior outcomes in the combined group may stem from complementary physiological mechanisms. HIIT enhances mitochondrial biogenesis and fatty acid oxidation, while controlled carbohydrate intake reduces postprandial glucose excursions and stabilizes hepatic lipid handling, synergistically improving metabolic flexibility (33, 34). In parallel, intermittent high-intensity bouts increase pulsatile shear stress, up regulating endothelial nitric oxide synthase and improving vasomotor function, whereas dietary quality and energy control dampen pro-inflammatory signaling that otherwise impairs NO bioavailability (33). Additionally, micronutrient supplementation (e.g., ferrous succinate) in the dietary plan may mitigate anemia risks a concern raised by Lien while supporting growth and exercise tolerance. The observed lipid profile improvements may reflect HIIT’s up regulation of lipoprotein lipase activity along with dietary restriction of saturated fats, together promoting lower LDL-C and triglycerides (35). This dual-pathway framework exercise to drive shear/mitochondrial cues; diet to normalize substrate flux and inflammation consistent with the breadth and magnitude of changes observed. Furthermore, methodological insights from tissue adaptation research in musculoskeletal rehabilitation—such as high-volume injection paradigms in Achilles tendinopathy—offer parallel models for understanding how repeated stimuli and loading cycles drive structural and functional change in biological tissues, complementing the cardiovascular improvements seen with exercise-based interventions (36).

### 4.4 Nuanced interpretation of the lipid profile

While LDL-C, TC, and TG improved in all groups—with the largest changes in the combined arm the within-group decreases in HDL-C warrant careful interpretation in children. Short-term weight loss and negative energy balance can transiently lower HDL-C despite overall cardio metabolic improvement, with HDL-C often recovering during weight-maintenance phases. In pediatric cohorts, assay variability, maturation status, and recent dietary intake can also influence HDL-C. Clinically, risk is more closely linked to non-HDL-C or ApoB than to HDL-C alone; the substantial reductions in LDL-C and TC, together with central adiposity loss, suggest that the net atherogenic burden likely declined. Nevertheless, future work should include non-HDL-C, ApoB, and TG/HDL-C ratio (and, where feasible, lipoprotein subfractionation) to more precisely characterize risk changes.

### 4.5 Clinical relevance and implementation

From a translational perspective, the combined intervention offers several practical advantages. First, HIIT’s time efficiency may improve adherence during school weeks, while dietitian-guided meal plans provide a scaffold for families to implement energy and nutrient targets at home. Second, the consistent improvements in waist circumference and VDC indices linked to cardio metabolic risk support the clinical value of integrating structured exercise with basic dietary regulation in pediatric weight-management programs. Embedding such protocols into school or community settings with periodic professional supervision could enhance scalability and equity of access. In support of intervention sustainability, evidence for training program effectiveness in athletic and youth-relevant populations further underscores the feasibility of structured exercise interventions for broader deployment (37).

### 4.6 Limitations and future directions

A key limitation is the absence of a true control group (no intervention), which precludes definitive conclusions about the interventions’ efficacy relative to natural changes. While randomization minimized baseline differences, future studies should include non-intervention controls to isolate treatment effects. Additionally, the 9-week duration limits insights into long-term sustainability, and the homogeneity of the sample (Chinese children aged 6–12) necessitates validation in diverse populations. Finally, although our outcome battery was comprehensive, the study was not powered *a priori* for between-group differences in all secondary endpoints, and the lipid panel did not include apolipoprotein measures. Despite these limitations, our findings underscore the clinical relevance of combining HIIT with dietary interventions for pediatric weight management. This approach not only addresses obesity but also mitigates cardiometabolic risks, offering a holistic strategy for improving children’s health. Future research should prioritize longer follow-up, inclusion of no-intervention controls, and mechanistic assessments (vascular stiffness, inflammatory and metabolic signaling, and, where feasible, microbiota) to refine dose, composition, and sequencing of exercise and diet. Pragmatic studies in school and community settings incorporating cost, adherence, and implementation outcomes will be essential to translate these benefits at population scale.

## 5 Conclusion

The 9-week randomized trial of overweight children, the combined intervention of high-intensity interval training (HIIT) plus dietary modification produced broader and larger benefits than either moderate-intensity continuous training (MICT) or HIIT alone. Relative to the single-modality arms, the combined approach yielded greater improvements in cardiac output, vasodilatation coefficient and flow-mediated vasodilation, with concomitant increases in nitric oxide and reductions in endothelin-1, indicating enhanced endothelial function. These vascular gains were accompanied by greater decreases in central adiposity (waist circumference), body mass index, and atherogenic lipids (LDL-C, total cholesterol, triglycerides). Taken together, the response profile across hemodynamic, endothelial, and compositional outcomes supports a synergistic effect of structured exercise and dietary regulation in pediatric weight management.

## Data Availability

The raw data supporting the conclusions of this article will be made available by the authors, without undue reservation.

## References

[1] Faienza MF Chiarito M Molina-Molina E Shanmugam H Lammert F Krawczyk M . Childhood obesity, cardiovascular and liver health: a growing epidemic with age. World J Pediatr. (2020) 16:438–45. doi: 10.1007/s12519-020-00341-9, PMID: 32020441 PMC7224053

[2] Steinberger J Daniels SR. Obesity, insulin resistance, diabetes, and cardiovascular risk in children: an American Heart Association scientific statement from the atherosclerosis, hypertension, and obesity in the young committee (council on cardiovascular disease in the young) and the diabetes committee (council on nutrition, physical activity, and metabolism). Circulation. (2003) 107:1448–53. doi: 10.1161/01.CIR.0000060923.07573.F2, PMID: 12642369

[3] Mihuta M. S. Paul C. Borlea A. Roi C. M. Pescari D. Stoian D. (2025). Childhood obesity: systemic complications, Early Atherosclerosis and the Value of Carotid Intima-Media Thickness. Beijing: People’s Medical Publishing House.

[4] Bendor CD Bardugo A Pinhas-Hamiel O Afek A Twig G. Cardiovascular morbidity, diabetes and cancer risk among children and adolescents with severe obesity. Cardiovasc Diabetol. (2020) 19:79. doi: 10.1186/s12933-020-01052-1, PMID: 32534575 PMC7293793

[5] Hiam L McKee M Dorling D. Influenza: cause or excuse? An analysis of flu's influence on worsening mortality trends in England and Wales, 2010–19. Br Med Bull. (2024) 149:72–89. doi: 10.1093/bmb/ldad028, PMID: 38224198 PMC10938544

[6] Franklin BA Eijsvogels TM Pandey A Quindry J Toth PP. Physical activity, cardiorespiratory fitness, and cardiovascular health: a clinical practice statement of the ASPC part I: bioenergetics, contemporary physical activity recommendations, benefits, risks, extreme exercise regimens, potential maladaptations. Am J Prev Cardiol. (2022) 12:100424. doi: 10.1016/j.ajpc.2022.100424, PMID: 36281324 PMC9586848

[7] Roca L Badric M Prskalo I. Macroregional differences in cardiorespiratory fitness in Croatian primary school children. Acta Kinesiol. (2023) 17:42–8. doi: 10.51371/issn.1840-2976.2023.17.2.7

[8] Calcaterra V Zuccotti G. Physical exercise as a non-pharmacological intervention for attenuating obesity-related complications in children and adolescents. Int J Environ Res Public Health. (2022) 19:5046. doi: 10.3390/ijerph19095046, PMID: 35564441 PMC9100328

[9] Roberts FL Markby GR. New insights into molecular mechanisms mediating adaptation to exercise; a review focusing on mitochondrial biogenesis, mitochondrial function, mitophagy and autophagy. Cells. (2021) 10:2639. doi: 10.3390/cells10102639, PMID: 34685618 PMC8533934

[10] Vettor R Valerio A Ragni M Trevellin E Granzotto M Olivieri M . Exercise training boosts eNOS-dependent mitochondrial biogenesis in mouse heart: role in adaptation of glucose metabolism. Am J Physiol Endocrinol Metab. (2014) 306:E519–28. doi: 10.1152/ajpendo.00617.2013, PMID: 24381004

[11] Cuddy TF Ramos JS Dalleck LC. Reduced exertion high-intensity interval training is more effective at improving cardiorespiratory fitness and cardiometabolic health than traditional moderate-intensity continuous training. Int J Environ Res Public Health. (2019) 16:483. doi: 10.3390/ijerph16030483, PMID: 30736402 PMC6388288

[12] Li QQ Qin KR Zhang W Guan XM Cheng M Wang YX. Advancements in the regulation of different-intensity exercise interventions on arterial endothelial function. Rev Cardiovasc Med. (2023) 24:306. doi: 10.31083/j.rcm2411306, PMID: 39076455 PMC11262454

[13] Fountaine CJ. SHAREABLE RESOURCE: ten training principles to guide exercise prescription. ACSMs Health Fit J. (2024) 28:88. doi: 10.1249/FIT.0000000000000986

[14] Wahl P Bloch W Proschinger S. The molecular signature of high-intensity training in the human body. Int J Sports Med. (2022) 43:195–205. doi: 10.1055/a-1551-9294, PMID: 34265857 PMC8885329

[15] Ross M Kargl CK Ferguson R Gavin TP Hellsten Y. Exercise-induced skeletal muscle angiogenesis: impact of age, sex, angiocrines and cellular mediators. Eur J Appl Physiol. (2023) 123:1415–32. doi: 10.1007/s00421-022-05128-6, PMID: 36715739 PMC10276083

[16] Zhang L Liu Y Sun Y Zhang X. Combined physical exercise and diet: regulation of gut microbiota to prevent and treat of metabolic disease: a review. Nutrients. (2022) 14:4774. doi: 10.3390/nu14224774, PMID: 36432462 PMC9699229

[17] Oliva F Bartoli A Garofalo E Calabrese M Oliva G Maffulli N. Influence of exercise on musculoskeletal disorders associated with gut microbiota: a narrative review. Muscles Ligaments Tendons J. (2023) 13:2–10. doi: 10.32098/mltj.01.2023.01

[18] Campbell SC Wisniewski PJ. Exercise is a novel promoter of intestinal health and microbial diversity. Exerc Sport Sci Rev. (2017) 45:41–7. doi: 10.1249/JES.0000000000000096, PMID: 27782912

[19] Nay K Jollet M Goustard B Baati N Vernus B Pontones M . Gut bacteria are critical for optimal muscle function: a potential link with glucose homeostasis. Am J Physiol Endocrinol Metab. (2019) 317:E158–71. doi: 10.1152/ajpendo.00521.2018, PMID: 31039010

[20] Moore DR. Nutrition to support recovery from endurance exercise: optimal carbohydrate and protein replacement. Curr Sports Med Rep. (2015) 14:294–300. doi: 10.1249/JSR.0000000000000180, PMID: 26166054

[21] Onu A Trofin DM Tutu A Onu I Galaction AI Sardaru DP . Integrative strategies for preventing and managing metabolic syndrome: the impact of exercise and diet on oxidative stress reduction—a review. Life. (2025) 15:757. doi: 10.3390/life15050757, PMID: 40430185 PMC12113156

[22] Paoli A. The influence of physical exercise, ketogenic diet, and time-restricted eating on De novo lipogenesis: a narrative review. Nutrients. (2025) 17:663. doi: 10.3390/nu17040663, PMID: 40004991 PMC11858292

[23] Lloyd RS Oliver JL. The youth physical development model: a new approach to long-term athletic development. Strength Cond J. (2012) 34:61–72. doi: 10.1519/SSC.0b013e31825760ea

[24] Kale M Yol Y Tolali AB Ayaz E. Effects of repetitive different jump pre-conditioning activities on post activity performance enhancement: effects of repetitive different jump preloads. Acta Kinesiol. (2023) 17:55–61. doi: 10.51371/issn.1840-2976.2023.17.2.9

[25] Eddolls WT McNarry MA Stratton G Winn CO Mackintosh KA. High-intensity interval training interventions in children and adolescents: a systematic review. Sports Med. (2017) 47:2363–74. doi: 10.1007/s40279-017-0753-8, PMID: 28643209 PMC5633633

[26] dos Santos Albarello JC Torres Laett C Soares de Palma AM Mandarino M Cavalcante da Silva S Goes RA . Associated ACL reconstruction and meniscal repair do not affect the evolution of isokinetic parameters in professional athletes: a prospective study with a one-year follow-up. Muscles Ligaments Tendons J. (2024) 14:450–7 doi: 10.32098/mltj.03.2024.08, PMID: 41176162

[27] Revision Committee of the Guidelines for the Prevention and Control of Childhood Obesity (2021) Guidelines for the prevention and control of childhood obesity. Beijing: People’s Medical Publishing House.

[28] Wewege M Van Den Berg R Ward RE Keech A. The effects of high-intensity interval training vs. moderate-intensity continuous training on body composition in overweight and obese adults: a systematic review and meta-analysis. Obes Rev. (2017) 18:635–46. doi: 10.1111/obr.12532, PMID: 28401638

[29] Moreau KL Clayton ZS DuBose LE Rosenberry R Seals DR. Effects of regular exercise on vascular function with aging: does sex matter? Am J Phys Heart Circ Phys. (2024) 326:H123–37. doi: 10.1152/ajpheart.00392.2023, PMID: 37921669 PMC11208002

[30] Sorop O Olver TD van de Wouw J Heinonen I van Duin RW Duncker DJ . The microcirculation: a key player in obesity-associated cardiovascular disease. Cardiovasc Res. (2017) 113:1035–45. doi: 10.1093/cvr/cvx093, PMID: 28482008

[31] Liu H Li J Gao T. Analysis of the intervention effect of diet and nutrition combined with exercise therapy on childhood obesity. Syst Med. (2023) 8:155–8. doi: 10.19368/j.cnki.2096-1782.2023.14.155

[32] Mu S Dang X Yang C. 24 w effect of aerobic exercise combined with modified low-carbon diet intervention on body composition and endothelial cell function in overweight/obese children. Chin Health Eng. (2022) 21:377–80. doi: 10.19937/j.issn.1671-4199.2022.03.007

[33] Wang X Fan M Meng Q Zhang D Liu T. Effects of high-intensity interval training on lipid metabolism, tumor necrosis factor-α, and C-reactive proteinin overweight children. Am J Transl Res. (2024) 16:6889–902. doi: 10.62347/LZIS9354, PMID: 39678562 PMC11645581

[34] Jahangiri M Shahrbanian S Gharakhanlou R. High intensity interval training alters gene expression linked to mitochondrial biogenesis and dynamics in high fat diet fed rats. Sci Rep. (2025) 15:5442. doi: 10.1038/s41598-025-86767-5, PMID: 39952980 PMC11828894

[35] Mambrini SP Grillo A Colosimo S Zarpellon F Pozzi G Furlan D . Diet and physical exercise as key players to tackle MASLD through improvement of insulin resistance and metabolic flexibility. Front Nutr. (2024) 11:1426551. doi: 10.3389/fnut.2024.1426551, PMID: 39229589 PMC11370663

[36] Hassan R Poku D Miah N Maffulli N. High-volume injections in Achilles tendinopathy: a systematic review. Br Med Bull. (2024) 152:35–47. doi: 10.1093/bmb/ldae015, PMID: 39496560

[37] Naderifar H Babakhanian S Najafi-Vosough R Karimizadeh Ardakani M. Neuromuscular control training is effective to prevent ankle sprains in athletes. Muscles Ligaments Tendons J. (2024) 14:188–96. doi: 10.32098/mltj.01.2024.16

